# Depression, anxiety, and stress among Ugandan university students during the COVID-19 lockdown: an online survey

**DOI:** 10.4314/ahs.v21i4.6

**Published:** 2021-12

**Authors:** Sarah Maria Najjuka, Gaudencia Checkwech, Ronald Olum, Scholastic Ashaba, Mark Mohan Kaggwa

**Affiliations:** 1 Makerere University, College of Health Sciences, Uganda, P.O. Box 7062, Kampala; 2 Mbarara University of Science and Technology, Department of Psychiatry, Uganda, P. O. Box 1410, Mbarara; 3 African Centre for Suicide Prevention and Research, Mbarara, Uganda

**Keywords:** COVID-19 lockdown, University students, Mental health

## Abstract

**Background:**

COVID19 pandemic forced most countries to lockdown, leading to the prolonged closure of many learning institutions. This dramatic shift led to increase of mental illness symptoms among university students.

**Objective:**

To determine the prevalence and factors associated with symptoms of depression, anxiety, and stress among Uganda's university students during the COVID-19 lockdown.

**Methods:**

We conducted a one-month online survey using the Depression Anxiety and Stress Scale (DASS-21).

**Results:**

Participants n=321 were enrolled with mean age, 24.8(SD=5.1) years and 198(61.7%) were males. The prevalence of mental health symptoms among participants was 80.7%, 98.4%, and 77.9% for depression, high levels of anxiety, and stress, respectively. Statistically significant association between mental health symptoms on multi-logistic regression was found with Males (depression=2.97[1.61–5.48] and stress=1.90[1.07–3.35]), engagement in leisure activity (depression= 1.87[1.01–3.49] and stress=1.98[1.10–3.56]), and being finalist (stress=0.55[0.31- 0.97]). Use of addictive substances seem to potentially alleviate symptoms of depression, anxiety and stress in the short term.

**Conclusions:**

The findings of this study suggest a high prevalence of symptoms of depression, anxiety and stress among university students during the COVID-19 lockdown. Students' mental health should be monitored by all stakeholders, especially as the pandemic progresses.

## Introduction

Coronavirus disease 2019 (COVID-19) is an acute respiratory disease caused by a novel human coronavirus (SARS-Cov-2); which was declared a pandemic by March 2020 1, 2. Its highly contagious nature prompted the implementation of strict infection control measures in countries that had reported cases of the disease 3. Before the first case of COVID-19 on 21 March 2020 in Uganda, a national lockdown similar to other African countries was declared, which involved the complete closure of learning-institutions to control the spread of the coronavirus 4–6. Nevertheless, the escalating mortality rates of COVID-19 globally led to the prolonged closure of schools and universities; that documented social and psychological effects including loss of hope, uncertainty about the future of education, loneliness, and social isolation among university students 7, 8.

Previous studies have reported increasing levels of depression, anxiety, and stress among university students as the COVID-19 pandemic persists^9^–^11^. One study conducted among university students in China between January 31^st^ and February 5^th^ 2020, found the prevalence of anxiety and depression symptoms at 7.7% and 12.2%, respectively 12. In another study carried out in Italy between 24 March and 3 May, it was observed that 34.3% and 27.8% of students had anxiety and depressive symptoms, respectively 13. In Bangladesh, the prevalence of depressive and anxiety symptoms were much higher among University students, 82.4% and 87.7%, respectively in a study conducted from May 6^th^ – 12^th^, 2020 14. Likewise, very high levels of stress have also been reported among university students around the globe during the pandemic^7^, ^15^, ^16^. A high prevalence of mental symptoms (depression, anxiety, and stress) among students is associated with higher rates of burnout, suicide, dropout, and poor academic performance^11^,^17^–^22^.

Living in urban areas, being from a family with a stable income, and students living with parents have been documented to be protective against anxiety, whereas having relatives or acquaintances with COVID-19 increases the risk for anxiety^23^. Also, the use of the official channels as the main source of information about COVID-19 is documented as a protective factor for both anxiety and depression. On the other hand, the fear of the risk of exposure was found to be significantly associated with a high risk of having mental illness symptoms 24, 25. Moreover, students who anticipated participating in events such as exchange programs and graduation ceremonies (finalists) were more stressed following the closure of institutions of learning 26. During the pandemic use of substances of addiction (alcohol, cannabis, tobacco, and prescription medication) increased drastically, especially among university students^27^–^29^. These substances of addiction have been associated with increased mental illness symptoms among university students during the pandemic 27–30. Other factors that have been identified to be associated with mental symptoms during the pandemic include gender (female), lack of interest towards studying, not having close friends, not having active leisure activities, family history of mental illness, and conflict with friends 14, 31, 32.

Despite the challenges of a national lockdown, including the mandate for social distancing and persistent closure of institutions of learning; information is limited about the mental health of Ugandan university students whose academic progress was halted. Specifically, there were no Ugandan universities that continued with online learning formats which resulted in an abrupt cessation of learning for all learners in the country as a way to prevent the spread of the COVID-19. This specific study aimed at screening for the mental illness symptoms of students by assessing symptoms of depression, anxiety, and stress during the COVID19 lockdown in Uganda.

## Methods

### Study design and procedure

Due to the increasing psychological changes associated with the pandemic, a one-month online survey from June 29, 2020, to July 29, 2020, was conducted. A semi-structured questionnaire using Google forms (https://forms.gle/nqfZCR55g9jLFSqV8), was developed, and posted in various WhatsApp groups of university students across the country. Respondents were recruited by a convenience sampling strategy focused on recruiting students all over the country during the COVID-19 lockdown. The students on the study group sent a link with this message “Hi, I am (name of student), a student at (university). As universities plan to reopen, the mental health of university students during the COVID-19 pandemic in Uganda should be known to inform adequate planning. Please help me take some time and fill in this form and send it to any other university students or university student groups”. Those who voluntarily accepted to participate clicked on the link to access the questionnaire. This method was preferred since the universities were closed, thereby excluding universities from the process of identifying students and distributing the survey. The design of the questionnaire permitted only eligible participants to participate in the study; that is, students 18-years and above.

### Ethical considerations

The study was approved by the Mulago Hospital Research and Ethics Committee under the reference number MHREC 1875. All participants provided informed consent.

### Study site and population

There are 9 public and 41 private universities in Uganda33. The study participants were part of approximately 692,490 students who were enrolled in universities from 2013 to 2019 and consisted of finalists (students in their last year of study, for instance, second year, third year, fourth year, or fifth years depending on the academic program), semi-finalists, international students, among others from various of faculties of the respective universities^33^.

### Sample size

The minimal sample size required to produce statistical power of 80% was calculated using Epi Info StatCalc for population surveys version 7.2.2.6. Using a population size of approximately 700,000 university students in Uganda as of 2019, expected frequency of depression as a primary outcome at 50%, acceptable margin of error of 5% and design effect of 1.0, the minimum calculated sample size was 382.

### Data collection

Data were collected using a comprehensive questionnaire that consisted of different sections. The first section included socio-demographic characteristics that included: age, sex, marital status, religion, current district of residence, number of people they are staying with and the relationships, university, level of study (undergraduate or postgraduate), year of study, and special groups [finalist, international students, semi-finalists, and those who stayed at university premises after lockdown], the form of sponsorship (self or government), current use of a substance of addiction (alcohol, cannabis, cigarettes), current employment status, sources of financial support during the lockdown, history of any mental, and chronic illness). The second part of the questionnaire inquired about COVID-19 related variables, which included the source of knowledge about COVID-19, previous testing for COVID-19, positive COVID-19 test results, and having a friend/relative/neighbour who had tested positive for COVID-19.

The third part of the questionnaire consisted of standardized Depression Anxiety and Stress Scale-21 (DASS-21) 34. The DASS-21 is a free scale that consists of three simple self-report scales designed to measure more than one symptom of the emotional states of depression, anxiety, and stress with each scale containing seven items and divided into subscales with similar content. All subscales are rated on a four-point Likert scale type ranging from 0 (never) to 3 (almost always). The ranges for Depression are, normal (0 – 9), mild (10 – 12), moderate (13 – 20), severe (21 – 27), and extremely severe (28 – 42); for Anxiety, normal (0 – 6), mild (7 – 9), moderate (10 – 14), severe (15 – 19), and extremely severe (20 – 42); and for Stress, normal (0 – 10), mild (11 – 18), moderate (19 – 26), severe (27 – 34), and extremely severe (35 – 42). The DASS-21 has been used in various COVID-19 mental illness studies all over the globe and has shown to be a valid and reliable scale for assessing these mental health symptoms of individuals during the COVID-19 pandemic^35^,^36^. Despite the scale not being used in Uganda before, it has been validated for use in other non-clinical groups in Africa (South Africa) and it has shown good construct validity, acceptability, discriminant validity, convergent validity and reliability^37^.

### Statistical analysis

Descriptive statistics were used to present socio-demographic data, using the aforementioned cutoffs, the prevalence of symptoms of depression, anxiety, and stress was calculated. Depression and anxiety were classified as present (mild to extremely severe) and absent (normal), while anxiety was classified as higher (severe and extremely severe) and lower levels (mild and moderate). To determine the factors associated with depression, stress, and higher levels of anxiety, bivariate and multivariate logistic regression analyses were performed, Odds Ratios (OR) and 95% confidence intervals (CI) were presented. For statistical significance, a P-level of less than 0.05 used.

## Results

### Demographic Characteristics

**Figure 1:**
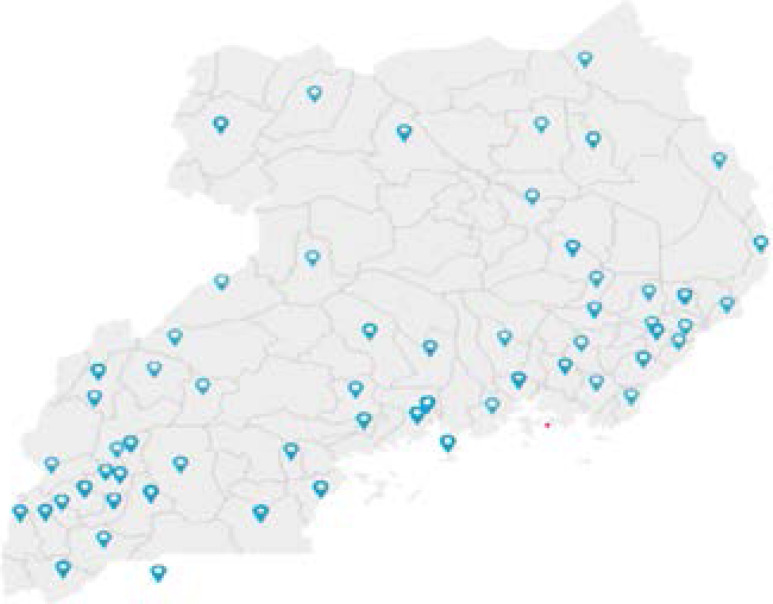
Maps of Uganda Showing the distribution of participants

Data were collected from a total of 321 eligible participants. The participants were from 15 Ugandan universities, coming from most regions of the country (figure 1).

Of the sample, 123 were females, mean (standard deviation (SD)) age 24.8 (5.1) years; 56 of the participants (17.5%) were married/cohabiting. The majority of the participants (69.8%) stayed in urban centres during the pandemic and 24.9% from the capital city Kampala (the most affected district in the country). The undergraduate students (262) were 81.6%; learners who had studied for three years represented 33.6% of the participants. Majority of the participants n = 169 (52.6%) were doing medical-related courses. Table 1 presents additional demographic and pandemic-related characteristics.

**Table 1:** Descriptive Statistics of Demographic Characteristics and Pandemic-Related Information for the Total Sample

Variable		N = 321
		n (%)
**Age**	24.8 (5.1)	
**Sex**	Female	123 (38.3)
	Male	198 (61.7)
**Religion**	Christian	283 (88.2)
	Islam	28 (8.7)
	Others	10 (3.1)
**Marital status**	Married/cohabiting	56 (17.5)
	Single	265 (82.6)
**Residence**	Rural	97 (30.2)
	Urban	224 (69.8)
**Education level**	Undergraduate	273 (86.4)
	Post and Graduate	43 (11.7)
**Year of study**	1	47 (14.6)
	2	87 (27.1)
	3	108 (33.6)
	4	60 (18.7)
	5	19 (5.9)
**Sponsorship**	Government	89 (27.7)
	Others	34 (10.6)
	Private	198 (61.7)
**Employment**	No	256 (79.8)
	Yes	65 (20.2)
**Finalist**	No	199 (62)
	Yes	122 (38)
**International students**	No	301 (93.8(
	Yes	20 (6.2)
**Stayed at university during closure**	No	288 (89.7)
	Yes	33 (10.3)
**Semi-finalist**	No	225 (70.1)
	Yes	96 (29.9)
**Number of people they currently live with [median (range)]**	14 (1 – 20)	
**People they stay with in lockdown**		
**1. Children**	No	281(87.5)
	Yes	40(12.5)
**2. Friend**	No	290(90.3)
	Yes	31(9.7)
**3. Parents**	No	191(59.5)
	Yes	130(40.5)
**4. Relatives**	No	160(49.8)
	Yes	161(50.2)
**5. Spouse**	No	282(87.9)
	Yes	39(12.2)
**6. Roommate**	No	312(97.2)
	Yes	9(2.8)
**Number of financial supporting sources**	0	32(10)
	1	250(77.9)
	2	35(10.9)
	3	4(1.2)
**Leisure activity involvement during lockdown**	No	179(55.8)
	Yes	142(47.2)
**History of mental illness**	Yes	290(90)
	No	31(10)
**History of chronic illness**	No	289(90)
	yes	32(10)
**Current use of a Substance of addiction**	No	256(80.2)
	Yes	63(19.8)
**1. Alcohol**	48 (15)	
**2. Caffeine**	17 (5.3)	
**3. Cannabis**	3 (0.9)	
**4. Cigarette**	2 (0.6)	
**5. Others**	2 (0.6)	

### Prevalence of symptoms of depression, anxiety, and stress

The prevalence of symptoms for the three mental health conditions among the total sample was 80.7% for depression (n = 259, including 35 [10.9%] with mild, 134 [41.7] with moderate, 90 [28.0%] with severe, and none with extremely severe depression). A total of 316 (98.4%) participants had symptoms of anxiety, including 11[3.4%] with mild and 59 [18.4] with moderate that is, low levels of anxiety, 80 [24.9] with severe and 166 participants [51.7%] with extremely severe anxiety that is, high levels of anxiety. For stress n = 250 (77.9%), including 120 [37.4%] with mild, 130 [40.5], no participants with severe and extremely severe stress.

### Factors associated with symptoms of depression, stress, and higher levels of anxiety

Table 2 presents the results of the unadjusted analysis of demographic and pandemic-related variables. In the multivariable analysis, factors associated with the symptoms of depression and stress were male sex, having a leisure activity during the lockdown, and the use of addictive substances. Using addictive substances was protective against higher levels of anxiety in the population (AOR, 0.50; 95% CI, 0.27 – 0.94).

**Table 2:** Bivariable Regression Analysis of Factors Associated with Symptoms of Depression, Anxiety, and Stress

Variable	Depression		Higher levels of Anxiety		Stress	
	OR (95%CI)	P value	OR (95%CI)	P value	OR (95%CI)	P value
**Age**	1.00 (0.94 – 1.06)	0.963	1.00 (0.95 – 1.06)	0.887	0.98 (0.94 – 1.04)	0.570
**Sex**						
Male	2.52 (1.43 -4.43)	0.001	1.60 (0.94 – 2.74)	0.086	1.66 (0.97 – 2.83)	0.062
Female	1 (reference)		1 (reference)		1 (reference)	
**Religion**						
Christian	0.44 (0.55 – 3.55)	0.441	0.38 (0.05 – 3.05)	0.362	0.89 (0.18 – 4.30)	0.886
Islam	0.67 (0.06 – 6.79)	0.732	0.41 (0.04 – 3.88)	0.435	0.75 (0.13 – 4.40)	0.750
Others	1 (reference)		1 (reference)		1 (reference)	
**Marital status**						
Single	1.02 (0.50 – 2.12)	0.945	1.14 (0.57 – 2.27)	0.709	1.22 (0.62 – 2.38)	0.568
Married/cohabiting	1 (reference)		1 (reference)		1 (reference)	
**Residence**						
Urban	1.12 (0.62 – 2.04)	0.697	1.49 (0.85 – 2.6)	0.165	1.46 (0.83 – 2.54)	0.185
Rural	1 (reference)		1 (reference)		1 (reference)	
**Education level**						
Undergraduate	1.09 (0.49 – 2.40)	0.837	0.78 (0.34 – 1.76)	0.548	1.20 (0.57 – 2.53)	0.620
Post and Graduate	1 (reference)		1 (reference)		1 (reference)	
**Year of study**						
1	1 (reference)		1 (reference)		1 (reference)	
2	0.46 (0.16 – 1.32)	0.148	0.81 (0.34 – 1.89)	0.624	1.14 (0.45 – 2.84)	0.784
3	0.59 (0.21 – 1.71)	0.336	1.07 (0.46 – 2.49)	0.869	0.75 (0.32 – 1.75)	0.501
4	0.30 (0.10 – 0.89)	0.030	1.21 (0.46 – 3.16)	0.694	0.65 (0.26 – 1.64)	0.364
5	0.45 (0.10 – 1.88)	0.273	0.72 (0.21 – 2.51)	0.609	0.66 (0.19 – 2.32)	0.521
**Sponsorships**						
Government	0.93 (0.50 – 1.72)	0.824	1.17 (0.63 – 2.20)	0.615	1.11 (0.61 – 2.04)	0.724
Others	2.62 (0.76 – 8.99)	0.127	0.80 (0.35 – 1.85)	0.608	1.41 (0.55 – 3.62)	0.472
Private	1 (reference)		1 (reference)		1 (reference)	
**Employment**						
Yes	1.07 (0.53 – 2.15)	0.845	1.15 (0.58 – 2.25)	0.692	1.17 (0.59 – 2.30)	0.645
No	1 (reference)		1 (reference)		1 (reference)	
**Finalist**						
Yes	0.54 (0.31 – 0.95)	0.032	0.60 (0.35 – 1.03)	0.989	0.51 (0.30 – 0.87)	0.013
No	1 (reference)		1 (reference)		1 (reference)	
**International students**						
Yes	1,38 (0.39 – 4.87)	0.615	0.84 (0.30 – 2.41)	0.752	0.84 (0.29 – 2.40)	0.749
No	1 (reference)		1 (reference)		1 (reference)	
**Stayed at university during closure**						
Yes	1.09 (0.43 – 2.76)	0.862	2.98 (0.88 – 10.11)	0.079	0.87 (0.38 – 2.03)	0.756
No	1 (reference)		1 (reference)		1 (reference)	
**Semi-finalist**						
Yes	1.05 (0.57 – 1.94)	0.867	1.77 (0.94 – 3.32)	0.077	1.47 (0.80 – 2.69)	0.215
No	1 (reference)		1 (reference)		1 (reference)	
**Number of people they currently** **live with**	0.99 (0.95 – 1.04)	0.714	0.99 (0.94 – 1.04)	0.655	0.99 (0.95 – 1.04)	0.807
**People they stay with in lockdown**						
**(1) Children**						
Yes	0.95 (0.41 – 2.18)	0.907	1.30 (0.55 – 3.09)	0.556	0.71 (0.34 – 1.51)	0.382
No	1 (reference)		1 (reference)		1 (reference)	
**(2) Friend**						
Yes	1.27 (0.47 – 3.46)	0.637	1.54 (0.57 -4.16)	0.398	2.03 (0.68 -6.0)	0.202
No	1 (reference)		1 (reference)		1 (reference)	
**(3) Parents**						
Yes	0.79 (0.45 – 1.38)	0.406	0.90 (0.52 – 1.54)	0.695	0.84 (0.50 – 1.44)	0.539
No	1 (reference)		1 (reference)		1 (reference)	
**(4) Relatives**						
Yes	0.44 (0.25 – 0.79)	0.006	0.76 (0.44 – 1.29)	0.307	0.50 (0.29 – 0.86)	0.012
**(5) Spouse**						
Yes	1.12 (0.46 – 2.64)	0.818	0.87 (0.39 – 1.94)	0.735	0.94 (0.42 – 2.08)	0.878
No	1 (reference)		1 (reference)		1 (reference)	
**(6) Roommate**						
Yes	1.94 (0.24 – 15.84)	0.534	0.56 (0.14 – 2.29)	0.419	0.99 (0,20 – 4.89)	0.994
No	1 (reference)		1 (reference)		1 (reference)	
**Financial supporting sources**						
Yes (1 – 3 sources)	0.40 (0.12 – 1.37)	0.145	1.09 (0.64 – 1.85)	0.746	1.19 (0.51 – 2.79)	0.679
No	1 (reference)		1 (reference)		1 (reference)	
**Leisure activity involvement during lockdown**						
Yes	1.87 (1.04 – 3.36)	0.036	1.33 (0.77 – 2.29)	0.302	1.89 (1.09 – 3.30)	0.024
No	1 (reference)		1 (reference)		1 (reference)	
**History of mental illness**						
Yes	1.19 (0.44 – 3.24)	0.736	0.56 (0.25 – 1.25)	0.158	0.62 (0.27 – 1.43)	0.265
No	1 (reference)		1 (reference)		1 (reference)	
**Current use of a Substance of addiction**						
Yes	0.26 (0.14 – 0.47)	0.001	0.50 (0.27 – 0.94)	0.030	0.27 (0.15 – 0.49)	0.001
No	1 (reference)		1 (reference)		1 (reference)	
**History of chronic illness**						
Yes	1.19 (0.44 – 3.24)	0.736	0.97 (0.40 – 2.36)	0.952	0.75 (0.32 – 1.76)	0.510
No	1 (reference)		1 (reference)		1 (reference)	
**Main source of information about COVID 19**						
Neighbours/peers/relatives	0.30 (0.05 – 1.94)	0.205	1.28 (0.14 – 12.1)	0.830	0.26 (0.05 – 1.45)	0.123
Newspapers	0.25 (0.05 – 1.28)	0.095	0.26 (0.06 – 1.19)	0.082	0.43 (0.09 – 2.06)	0.289
Social media	0.53 (0.21 – 1.33)	0.177	0.78 (0.35 – 1.72)	0.535	0.82 (0.37–1.82)	0.624
Television	0.66 (0.25 – 1.70)	0.388	0.98 (0.43 – 2.26)	0.968	1.13 (0.48 – 2.63)	0.779
WHO/CDC platforms	1.12 (0.35 – 3.58)	0.852	1.19 (0.45 – 3.18)	0.723	1.38 (0.50 – 3.75)	0.534
Ministry of health platform	1 (reference)		1 (reference)		1 (reference)	
**Having ever tested for COVID 19**						
Yes	2.74 (0.63 – 11.97)	0.180	1.15 (0.42 – 3.18)	0.787	7.04 (0.93 – 53.12)	0.058
No	1 (reference)		1 (reference)		1 (reference)	
**Having a relative/friend/neighbour who tested positive**						
Yes	0.43 (0.14 – 1.30)	0.135	0.77 (0.24 – 2.50)	0.667	0.73 (0.22 – 2.36)	0.597
No	1 (reference)		1 (reference)		1 (reference)	

Males had at least twice the likelihood of having symptoms of depression and stress than females (AOR; 2.97 95% CI, 1.61 – 5.48 for depression; AOR, 1.90; 95% CI, 1.07 – 3.35 for stress). Finalists were less likely to have symptoms of depression and stress (AOR, 0.62; 95% CI, 0.34 – 1.12) and (AOR, 0.55; 95% CI, 0.31 – 0.97 for stress), respectively compared to the non-finalists. Individuals who had a leisure activity during the lockdown had an increased likelihood of having symptoms of depression and stress (AOR, 1.87; 95% CI, 1.01 – 3.49 for depression; AOR, 1.98; 95% CI, 1.10 – 3.56 for stress). Using addictive substances was protective against all mental symptoms (AOR, 0.50; 95% CI, 0.27 - 0.94 for higher levels of anxiety; AOR, 0.24; 95% CI, 0.13 – 0.47 for depression; AOR, 0.27; 95% CI, 0.14 – 0.51 for stress). The detailed results of the multivariable analysis are shown in Table 3.

**Table 3:** Multivariable Regression Analysis of Factors Associated with Symptoms of Depression, Anxiety, and Stress

Variable	Depression		Stress	
	AOR	P value	AOR	P value
**Sex**				
Male	2.97 (1.61 – 5.48)	0.001	1.90 (1.07 – 3.35)	0.028
Female	1 (reference)		1 (reference)	
**Finalist**				
Yes	0.62 (0.34 – 1.12)	0.116	0.55 (0.31 – 0.97)	0.038
No	1 (reference)		1 (reference)	
**Leisure activity involvement during lockdown**				
Yes	1.87 (1.01 – 3.49)	0.047	1.98 (1.10 – 3.56)	0.023
No	1 (reference)		1 (reference)	
**Substance use**				
Yes	0.24 (0.13 – 0.47)	<0.001	0.27 (0.14 – 0.51)	<0.001
No	1 (reference)		1 (reference)	

## Discussion

This study investigated the prevalence and associated factors of symptoms of depression, anxiety, and stress among Ugandan University students during the lockdown, when learning was precipitously halted as a measure to reduce COVID 19 spread. The prevalence's of these mental symptoms were high, with three-quarters of the sample having symptoms; anxiety was highest, followed by depression, and then stress. Using addictive substances and being a finalist reduced the likelihood of having these mental symptoms. However, having a leisure activity during the lockdown and male sex were more associated with symptoms of stress and depression.

Almost all participants had severe levels of anxiety, indicating fear during this period mostly due to COVID-19 and fear of the unknown future; possibly, attributed to the high levels of misinformation ongoing during the pandemic^38^. The study findings are similar to a study done in a similar study group in Bangladeshi University students in May of 2020, with high prevalence symptoms of depression (82.4%) and anxiety (87.7%) (14). However, a systematic review of these mental symptoms in the general population with results published before May 2020 (early stages of the pandemic) indicated lower levels of stress (29.6%), anxiety (31.9%), and depression (33.7%) 39. This could be due to an increasing rate of mental symptoms in the general population, leading to a predicted pandemic of mental illness^9^, which other researchers have also predicted due to increasing mental symptoms as the pandemic progresses 10. This calls for more emphasis on students' mental health as the pandemic continues.

Male sex was associated with having symptoms of depression and stress, similar to a general population study during the pandemic that found more stressed and depressed males 40. This was a surprising phenomenon seen in the pandemic because in Ugandan culture, with a notation of “men don't cry”; makes men not to typically open up about their mental symptoms as this is seen as a sign of weakness. This reporting could be attributed to the tool that was diverse and explored many symptoms leading to unmasking of the true mental symptoms experienced by these participants. With more males statistically having been more infected by COVID-19; this potentially puts them at high stress and fear of getting infected, hence the high levels of stress^10^, ^41^. Male students have also been found to not adhere to quarantine and protective measures as compared to females 38, putting them at higher risk, thus, more fear and mental unwellness. Additionally, Uganda men, coping mechanisms for mental illness were eliminated during the pandemic such as freedom to move around, socialize, commune around shared interests, have agency, autonomy, among others. We suggest that, during the pandemic, more special attention should be given to male students given their poor mental health-seeking practices 42. Culturally, males are more likely to engaged in substance abuse 43.

Despite men having higher odds of mental symptoms; using substances of addiction such as alcohol in this study, reduced the likelihood to experience these symptoms. Could this paradoxical phenomenon of substance of addiction be a mode of self-medication to the symptoms experienced? as shown by previous studies, that these substances relieve depression and anxiety 44, 45. Many substances of addiction have a similar mode of action as antidepressants and anxiolytics, thereby potentially treating or resolving the symptoms of anxiety, depression, or stress these individuals had or could have had. More university students would be engaged in abuse/use of these substances due to high levels of boredom during the pandemic. With such high levels of mental illness symptoms, we could be having an increase in the number of dependences on addictive substances as the pandemic progresses, that is. the second pandemic of mental illness deliberating the most promising age and a group of the near future (university students).

Individuals who had any kind of leisure activity were also at a higher risk of stress and depression. This may be attributed to using them as a means of treating their mental symptoms. Previous research has indicated leisure activities such as exercise, yoga, watching movies, and singing can be used in the treatment of depression and stress 46. Other studies have also shown that being involved in leisure activities lowers preventive behaviours thus putting them at more risk of getting COVID-19, thus more anxiety then stress and depression 47.

Finalists had better mental health than other students, due to the government promising them an earlier resumption of schooling than other students since they were considered an essential group 48. Thus, reducing fear of the unknown future among them. This was contradictory to previous a previous study that suggested these individuals to have higher levels of mental symptoms 26. The remaining Ugandan students may be in great fear and affected with great boredom hence higher levels, so engaging them in online learning may be the solution to combat these symptoms. Studies during the pandemic indicate that engaging students online comes with more advantages, such as, easy illustration, direct sharing with teachers, and less stressing due to each student learning at their own pace 49.

## Strength and limitations

The sample and study design may have influenced the proportions and the finding in the study. The major strength of our study was also conducted 4 months after lockdown of universities, a period that was full of uncertainty about going back to school or any linkage to their institutions, hence, more mental challenges, thus, the findings are a true representation of students' mental health challenges related to COVID-19.

Our study also had several limitations. First, this was an online survey using a convenience sampling method conducted in a period characterised by financial challenges and loss of employment opportunities hence we could have missed views of several students who were not able to access the internet and social media during the study period. Secondly, it involved self-reporting, thus leading to reporting and recall bias. Thirdly, the snapshot nature of the study limits our ability to determine the cause-effect relationship between different independent and outcome variables. We could not determine the true response rate based on the method we used to distribute the link (WhatsApp) and the google forms also do not track the number of individuals who view the link; thus, the recipients may not easily be determined. We cannot also guarantee that all the respondents are truly University students since the link was open to anyone who could access it. Lastly, we did not reach the desired minimum sample size after one month of data collection to ascertain sufficient power for the study and with half of the participants being medical students, hence making the results not generalisable. Despite these limitations, this was the available option since students were so dispersed and the universities were completely shut down.

## Conclusion

The present study shows that more than three-quarters of university students are having mental symptoms of anxiety, depression, and stress during the COVID-19 to lock down their institutes because of the pandemic and the prevailing pandemic. Therefore, there is a need for interventions to support youth with more opportunities of engagement in productive activities to avoid participating in risky leisure activities or redundancy which increases their risk of having mental illness symptoms.
